# Nausea as a non-motor fluctuation in Parkinson’s disease

**DOI:** 10.1016/j.prdoa.2022.100178

**Published:** 2022-12-20

**Authors:** Emily Weisbach, Joseph Friedman, Umer Akbar

**Affiliations:** a Neurology, Brown University Warren Alpert Medical School, Providence, RI, USA; b Movement Disorders Program, Butler Hospital, Providence, RI, USA

Non-motor symptom (NMS) fluctuations are common in Parkinson’s disease (PD), but despite their deleterious impact on quality of life, they are underrecognized. NMS fluctuations including problems with mood, cognition, fatigue, autonomic function and sleep, have been shown to occur in PD often, but not always, in conjunction with motor fluctuations [1], [2], [3]. We report a PD patient with predictable and recurrent nausea, which was responsive to her routine dose of levodopa, and propose it is a NMS due to wearing-off. Nausea is a common complaint in PD in the context of medication side effects or delayed gastric emptying, however, nausea as a fluctuating NMS is rarely mentioned in the literature, and not described in a case report [2], [4], [5].

Our patient is a 64-year-old woman with a 5-year history of PD and a 20-year history of Multiple Sclerosis (MS) who developed new onset periodic nausea associated with her PD medication. She had been taking carbidopa/levodopa for approximately 3 years prior to the onset of nausea and was taking carbidopa/levodopa 25/100 four times daily, four hours apart, when the nausea began, in addition to gabapentin, ocrelizumab, simvastatin and vitamins. She experienced nausea approximately 3–3.5 hours after and 30–60 min prior to each of her carbidopa/levodopa doses, as well as in the middle of the night. The nausea improved about 30 min after she took her next dose. Her pattern of nausea was reproducible almost every day and continued even when she added a fifth nighttime dose and shifted her doses an hour earlier, still four hours apart. She did not experience any significant motor off symptoms during her periods of nausea.

The patient had also tried over-the-counter anti-acids and “motion sickness pills” without effect. She had an unrevealing physical, a negative *H. pylori* test and a negative abdominal ultrasound with her primary doctor. Omeprazole and famotidine did not help. She was evaluated by a gastroenterologist who felt her nausea was likely of a central etiology and that an endoscopy was not warranted. In discussing other possibilities, the patient endorsed recent onset anxiety, but did not think she was ever nauseated and anxious at the same time. She also reported constipation that resolved with OTC medications. Her MS was well controlled on ocrelizumab and she stated that she never had nausea in the course of either MS or PD. She had a contrast-enhanced MRI after the onset of her nausea which showed known MS lesions, including one in the right brachium pontis, but no new lesions which could explain her new nausea.

Although initially hesitant to increase her carbidopa/levodopa dose or trial a dopamine extender due to medication sensitivity and previous side effects, about a year after nausea onset, her carbidopa/levodopa dosing was increased to six times daily, 1 tab every 3 hours, in addition to one dose at night. This resulted in near-complete resolution of her nausea.

The differential diagnosis of our patient’s nausea includes a fluctuating NMS, nausea related to recent-onset anxiety and a new area postrema demyelinating lesion, which was ruled out by her MRI. We believe that the nausea is most likely a NMS of her PD. Nausea caused by anxiety is unlikely as our patient denied ever feeling simultaneous nausea and anxiety. The nausea’s reproducibility in relation to her daily carbidopa/levodopa doses, the fact that the timing of her nausea shifted when she shifted her dose timing, and the significant improvement with moving her doses from four hours apart to three hours apart is compelling evidence that it is a non-motor symptom related to her carbidopa/levodopa schedule.

Ethical compliance statement

An IRB review was not required for this case report. Written consent to publish this case report was obtained from the patient. All authors have read and agree to the ethical compliance statement of this journal.

## Author contributions

- Emily Weisbach, MD is the primary author or this paper and contributed by completing an in depth interview with the patient about her symptoms and writing and editing the case report. Emily takes responsibility for the conduct of this research.
- Joseph Friedman, MD made suggestions on improving content for this article and edited it.
- Umer Akbar, MD suggested the writing of this article and contributed to content and editing of the paper.
Author disclosures

Funding sources and conflict of interest

- Emily Weisbach, MD: no relevant disclosures.
- Joseph Friedman, MD: no relevant disclosures.
- Umer Akbar, MD: no relevant disclosures.

Financial Disclosures

- Emily Weisbach, MD: no relevant disclosures.
- Joseph Friedman, MD: Royalties: Cambridge University, Karger Press, and Medlink. Consultant fees from *EPI*-Q.
- Umer Akbar, MD: no relevant disclosures.

## Declaration of Competing Interest

The authors declare that they have no known competing financial interests or personal relationships that could have appeared to influence the work reported in this paper.
